# A comment on: “Conventional and Microwave Hydrothermal Synthesis and Application of Functional Materials: A Review”

**DOI:** 10.3390/ma12213631

**Published:** 2019-11-05

**Authors:** Behrouz Jalouli, Aref Abbasi, S. Mohammad Musavi Khoei

**Affiliations:** 1 Department of Mining and Metallurgy, Amirkabir University of Technology, Tehran 1591634311, Iran; behrouzjalouli@aut.ac.ir; 2 School of Metallurgy and Materials Engineering, Iran University of Science and Technology (IUST), Narmak, Tehran 1684613114, Iran; aref_abasi@aut.ac.ir

**Keywords:** hydrothermal synthesis, solubility variations, growth mechanism, supersaturation

## Abstract

In the recent paper published in Materials (Yang et al., 2019), there is a mistake in the explanation of crystal growth mechanism of the hydrothermal method. The error in this article is discussed in this short communication.

This short paper deals with an error in the article published by Yang et al. [1]. The mechanism of crystalline growth under hydrothermal condition claimed in this paper is incorrect. Yang et al indicated a five-step mechanism for hydrothermal synthesis process. They expressed that in the second step “the ions or molecules are separated by the temperature difference between the upper and lower portions of the kettle. The ions or molecular groups are transported to the low-temperature region, where the seed crystal is grown to form a supersaturated solution” [1].

The two items below about supersaturation formation, nucleation and growth in hydrothermal process are expressed incorrectly:

1. Seed crystals growth forms supersaturation.
2. Low-temperature region is the place of nucleation and growth.

In fact, in the hydrothermal synthesis of nanoparticles, solubility plays the major role of precipitation. 

Ions solubility is a function of water dielectric constant [2]. Since the water dielectric constant decreases with rising temperature, solubility in high temperature reduces and causes supersaturation [3,4]. This supersaturation acts as the driving force of nucleation and growth and results in the nanoparticles precipitation [5]. Thus, it is better to say:

1. Solubility reduction is the major reason of supersaturation, and it causes seed crystal growth.
2. High temperature is better for nucleation and growth.
